# Genetic mapping and candidate gene identification of *BoGL5*, a gene essential for cuticular wax biosynthesis in broccoli

**DOI:** 10.1186/s12864-021-08143-7

**Published:** 2021-11-10

**Authors:** Fengqing Han, Jingjing Huang, Qi Xie, Yumei Liu, Zhiyuan Fang, Limei Yang, Mu Zhuang, Yangyong Zhang, Honghao Lv, Yong Wang, Jialei Ji, Zhansheng Li

**Affiliations:** Institute of Vegetables and Flowers, Chinese Academy of Agricultural Sciences, Key Laboratory of Biology and Genetic Improvement of Horticultural Crops, Ministry of Agriculture, #12 Zhong Guan Cun Nandajie Street, Beijing, 100081 China

**Keywords:** Cuticular wax, Broccoli, Fine mapping, Candidate gene, Functional analysis

## Abstract

**Background:**

The aerial organs of most terrestrial plants are covered by cuticular waxes, which impart plants a glaucous appearance and play important roles in protecting against various biotic and abiotic stresses. Despite many glossy green (wax-defective) mutants being well characterized in model plants, little is known about the genetic basis of glossy green mutant in broccoli.

**Results:**

B156 is a spontaneous broccoli mutant showing a glossy green phenotype. Detection by scanning electron microscopy (SEM) and chromatography-mass spectrometry (GC-MS) revealed that B156 is a cuticular wax-defective mutant, lacking waxes mostly longer than C28. Inheritance analysis revealed that this trait was controlled by a single recessive gene, *BoGL5*. Whole-genome InDel markers were developed, and a segregating F_2_ population was constructed to map *BoGL5*. Ultimately, *BoGL5* was mapped to a 94.1 kb interval on C01. The *BoCER2* gene, which is homologous to the *Arabidopsis CER2* gene, was identified as a candidate of *BoGL5* from the target interval. Sequence analyses revealed that *Bocer2* in B156 harbored a G-to-T SNP mutation at the 485th nucleotide of the CDS, resulting in a W-to-L transition at the 162nd amino acid, a conserved site adjacent to an HXXXD motif of the deduced protein sequence. Expression analysis revealed that *BoCER2* was significantly down-regulated in the leaves, stems, and siliques of B156 mutant than that of B3. Last, ectopic expression of *BoCER2* in *A. thaliana* could, whereas *Bocer2* could not, rescue the phenotype of *cer2* mutant.

**Conclusions:**

Overall, this study mapped the locus determining glossy phenotype of B156 and proved *BoCER2* is functional gene involved in cuticular wax biosynthesis which would promotes the utilization of *BoCER2* to enhance plant resistance to biotic and abiotic stresses, and breeding of *B. oleracea* cultivars with glossy traits.

**Supplementary Information:**

The online version contains supplementary material available at 10.1186/s12864-021-08143-7.

## Background

The surfaces of terrestrial plants are covered by cuticles that serve as protective barriers between plants and the environment [1]. The cuticle consists of cutin, polysaccharides and cuticular waxes [2] and plays important roles in protecting plants against water loss, ultraviolet radiation, deposits of pollutants, and both bacterial and fungal pathogens [1]. In addition, the cuticle also affects plant-insect interactions [3].

Plant cuticular waxes comprise very long chain fatty acids (VLCFAs) and their derivatives, including C24 to C32 alkanes, aldehydes, alcohols, ketones and esters [4]. In the endoplasmic reticulum (ER) of plant epidermal cells, multienzymatic fatty acid elongase (FAE) complexes [5] catalyze elongation by adding two carbons per elongation cycle, C16 and C18 acyl-CoA precursors. VLCFA derivatives are produced in two ways: most wax compounds, including aldehydes, alkanes, secondary alcohols, and ketones, are biosynthesized via the alkane-forming pathway, whereas alcohols and esters are biosynthesized via the alcohol-forming pathway [6].

In *Arabidopsis thaliana*, many wax-biosynthesis genes have been identified, including the FAE complex genes *KCS1*, *KCS2*, *KCS6*, *KCS9*, *KCS20* and *KCR1* [7, 8]; the VLCFA modification genes *CER4*, *MAH1*, *CER1*, *CER3*, *CER2* (−like) and *CER26* [9–11]; the transporter genes *DESPERADO*/*AtWBC11*, ABCG transporters, and the *CER5* gene [12, 13]; and the regulatory genes *WIN1*/*SHN1*, *SHN2*, *SHN3*, *MYB30*, *MYB41*, *CFL1*, *CER7*, and *CER9* [14, 15]. Mutants of these wax-related genes show a decrease or lack of cuticular waxes, resulting in glossy phenotypes.

Among the wax-deficient mutants, *cer2* shows glossy green appearance in stems and siliques due to the decrease of all waxes longer than C28 [16–18]. *CER2* gene has been mapped to *At4g24510* via chromosome walking and T-DNA insertion identification, which is expressed in aerial plant tissues, with higher level in stems and siliques, and trace level in leaves [17, 18]. *CER2* encodes an endoplasmic reticulum-localized protein homologous to BAHD acyltransferase, responsible for elongation of VLCFAs longer than C28, but unlike a typical BAHD acyltransferase, the CER2 protein localize exclusively to the nucleus, and the catalytic acyltransferase motif was not conserved and not required for its function [10].

Mutants showing glossy phenotypes have also been identified in various Brassica species, including *B.oleracea* [19–22], *B. napus* [23], and *B. rapa* [24]. In cabbage, 3 glossy mutants have been reported. Liu et al. identified a dominant glossy green cabbage mutant, *10Q*-*974gl*, and mapped the target gene *BoGL1* to C08, between marker SSRC08–76 (41.51 Mb) and the end of the chromosome; two tandem genes, *Bol018503* and *Bol018504*, which are homologous to *CER1* in *Arabidopsis*, were determined to be candidates for *BoGL1* [20]. Liu et al. reported a recessive glossy green cabbage mutant, *10Q-961*, and mapped the target gene *Cgl1* to a 188.7 kb interval at the end of C08; interestingly, *CER1* was also predicted as the candidate [21]. Gene editing by CRISPR/Cas9 systems further confirmed the essential role of *BoCER1* in synthesis of cuticular wax [25]. Liu et al. reported other recessive cabbage mutants (*HUAYOU2* and *LD10GL*) and identified *Bol013612* (which is homologous to *CER4*) as the candidate [19, 21]. The ectopic expression of *Bol013612* in *Arabidopsis* rescued the wax deficiency of *cer4* mutants [19].

Broccoli (*Brassica oleracea* L. var. *italica*), a subspecies of *B. oleracea*, is an important vegetable crop widely cultivated worldwide. Previously, researchers have reported glossy mutants of broccoli [26, 27], but no genetic studies on these plants have been conducted. In this study, we identified a novel spontaneous glossy mutant B156, of broccoli. Using an F_2_ population, we mapped the target gene *BoGL5* to a 94 kb. Subsequent gene prediction, sequencing and functional analyses suggested that *BoCER2* was a highly plausible candidate gene for *BoGL5*.

## Results

### Phenotype and inheritance of the glossy traits of the B156 plants

**Fig. 1:**
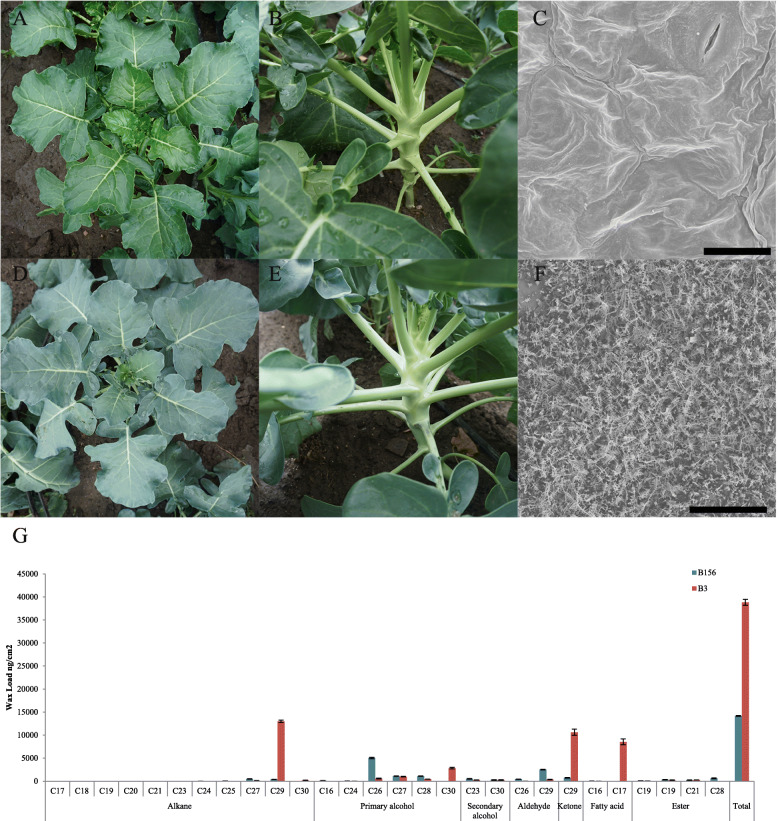
Phenotypes and wax loads of the mutant B156 and the wild-type B3 plants. B156 showed glossy appearance (**a**, **b**) in contrast with glaucous appearance of B3 (**d**, **e**). Scanning electron microscopy revealed dramatically reduced waxes on leaf of B156 (**c**) comparing with B3 (**f**), bar = 150 μm. Chromatography-mass spectrometry revealed total waxes and most > C28 components were significantly decreased in B156 (**g**)

In comparison with the glaucous appearance of wild-type plants B3 (Fig. 1D and E), the outer surfaces of B156, including the stems, leaves and siliques, showed glossy traits (Fig. 1A and B), possibly due to reduced load of cuticular waxes. Its consumed production, i.e. the flowering inflorescence (head and stalk) also became glossy green (Fig. S1). Other than the glossy appearance, the mutant showed no difference to wild type plants. The glossy phenotype of the B156 plants resembled the reported cabbage glossy mutants *10Q-974gl*, *HUAYOU2* and *LD10GL* [19, 21], but unlike that of *10Q-974gl*, its male fertility was not changed.

Observation by scanning electron microscopy (SEM) revealed that B3 leaves were covered with rod-shaped wax crystals, whereas B156 leaves were nearly devoid of wax crystals (Fig. 1C and F). Analysis of cuticular wax load and composition by GC-MS revealed a 63.5% reduction in total wax load of B156 mutant. All waxes longer than C28, except for C29 aldehydes, were dramatically decreased; in contrast C26, C27 and C28 waxes were slightly increased (Fig. 1G), indicating elongation of C28 VLCFA was blocked in B156 mutant.

### Fine mapping of the *BoGL5* gene

B156 was crossed with B3, a wild-type broccoli inbred line, and all the derived F_1_ plants showed a normal phenotype. The F_2_ plants segregated as 3:1 (1296 normal plants:412 glossy plants; χ^2^ = 0.70 < χ^2^_0.05,1_ = 3.84) according to a χ^2^ test, indicating that this glossy trait is controlled by a single recessive gene, *BoGL5*.

Primarily, ninety-three InDel primers covering all 9 chromosomes were designed to detect polymorphisms between the parents (B156 and B3) and the two DNA pools (the G pool and NG pool) (Table S1). A total of 61 primers showed polymorphisms between the parents, but only three markers, Broc2 (C01: 8,156,135), Broc3 (C01: 13,116,351) and Broc4 (C01: 18,102,957), were polymorphic between the two DNA pools (Table S1). A total of 89 randomly selected glossy F_2_ individuals were used to confirm the linkage and estimate the genetic distances. Broc2 and Broc3 were upstream of *BoGL5*, with genetic distances of 7.8 cM and 1.1 cM, respectively; Broc4 was downstream of *BoGL5*, with a genetic distance of 2.2 cM.

**Fig. 2:**
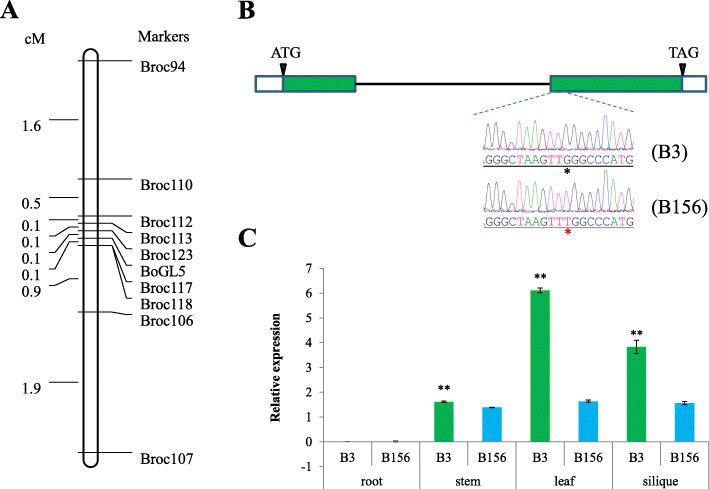
Linkage map and candidate gene analyses. *BoGL5* was mapped to an interval between Broc123 and Broc117 on chromosome C01 (**a**). Gene structure and allelic variation of the candidate gene *BoCER2* (**b**). *BoCER2* was significantly downregulated in the stems, leaves and siliques of B156 compared with those of B3 (**c**)

To narrow the candidate mapping region, 32 InDel markers within the 10 Mb genomic region were designed between Broc2 and Broc4. Ultimately, we obtained 21 markers linked to *BoGL5*. Two markers, Broc94 (C01: 11,005,498) and Broc4 (C01: 18,102,957), were used to genotype all 412 glossy F_2_ individuals to detect recombinants. As a result, we detected 20 recombinant individuals for Broc94 and 46 for Broc4 (Table S2). All recombinant individuals were further genotyped using eight markers located between Broc94 and Broc4 (Table S2). Ultimately, we mapped *BoGL5* to a 94.1 kb genomic region flanked by markers Broc123 and Broc117, each with a genetic distance of 0.12 cM. A genetic map is shown in Fig. 2A.

### *BolC1t01909H* (*BoCER2*) is a strong candidate for *BoGL5*

According to the gene prediction information in the HDEM reference genome, fifteen genes were located within the 94.1 kb mapping region (Table 1). Considering the cuticular waxes defects of B156, the causal gene should be structural, transporter or regulator gene associated with biosynthesis of VLCFAs and their derivatives. Among the fifteen genes, only one named *BolC1t01909H* was related to the biosynthesis of plant cuticular waxes. *BolC1t01909H* is predicted to encode an HXXXD-type acyltransferase family protein required for C28-to-C30 fatty acid elongation, consistent with the blocked elongation of C28 VLCFA and 63.5% total wax load reduction in B156 mutant. Additionally, *BolC1t01909H* showed nucleotide variation and differential expression levels between B156 and B3 plants (see the following results). Based on information of gene annotations, wax loads, gene sequencing and relative expression level results, we tentatively designated *BolC1t01909H* (*BoCER2*) as the candidate gene for *BoGL5*.

**Table 1:** Predicted genes in the target genomic region

Gene ID	Start position	Stop position	Homologous gene in *A. thaliana*	Annotation
BolC1t01905H	12,808,460	12,810,897	AT4G24460	CRT (chloroquine-resistance transporter)-like transporter 2
BolC1t01906H	12,811,839	12,813,742	AT4G24470	GATA-type zinc finger protein with a TIFY domain
BolC1t01907H	12,835,665	12,839,913	AT4G24480	Protein kinase superfamily protein
BolC1t01908H	12,841,073	12,845,010	AT4G24490	Hydroxyproline-rich glycoprotein family protein
BolC1t01909H	12,845,188	12,849,854	AT4G24510	HXXXD-type acyltransferase family protein
BolC1t01910H	12,856,294	12,860,159	AT4G24520	P450 reductase 1
BolC1t01911H	12,861,674	12,865,576	–	NADPH-cytochrome P450 reductase 1-like
BolC1t01912H	12,866,709	12,870,028	AT4G24530	O-fucosyltransferase family protein
BolC1t01913H	12,870,641	12,873,198	AT4G24540	AGAMOUS-like 24
BolC1t01914H	12,874,770	12,875,093	–	Polyadenylate-binding protein-interacting protein 6-like
BolC1t01915H	12,878,887	12,879,195	–	Profilin-1-like
BolC1t01916H	12,886,194	12,889,176	AT4G24550	Clathrin adaptor complexes medium subunit family protein
BolC1t01917H	12,889,708	12,894,099	AT4G24560	ubiquitin-specific protease 16
BolC1t01918H	12,899,929	12,900,876	AT4G24570	dicarboxylate carrier 2
BolC1t01919H	12,901,237	12,907,174	AT4G24580	Rho GTPase activation protein (RhoGAP) with a PH domain

### *Bocer2* of B156 mutant harbored a SNP that disrupted a conserved site adjacent to the HXXXD motif

We retrieved the gene sequence of *BolC1t01909H* (*BoCER2*) from the HDEM reference genome, the transcript structure of which seemed to be wrongly predicated. The full-length transcript of *BolC1t01909H* was obtained from an unpublished PacBio-based [PacBio RSII (Pacific Biosciences, Menlo Park, CA, USA)] RNA-seq dataset and amplified from B156 and B3 plants. The results revealed that the coding sequence of *BoCER2* is 1254 nt in length and comprised two exons (Fig. 2B). The deduced BoCER2 protein shared 74% identity with *Arabidopsis* CER2.

**Fig. 3:**
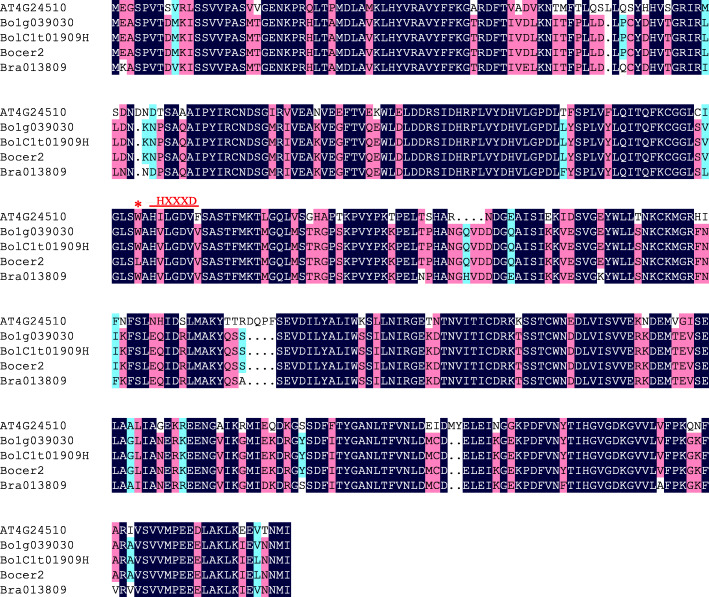
Protein sequence alignment of BoCER2 and its relatives. AT4G24510, CER2 of *A. thaliana*; Bo1g039010, BoCER2 in TO1000 (Chinese kale); BolC1t01909H, BoCER2 in B3 and HDEM (broccoli); Bocer2, mutant-type BoCER2 in B156; Bra013809, BraCER2 of *B. rapa*. The 162nd amino acid is indicated by the red asterisk

To determine whether *BoCER2* had nucleotide variation between B156 and B3, the 2 kb promoter region and 2462 bp gene body region were amplified from the genomic DNA of B156 and B3 plants. The *BoCER2* transcript was amplified from the cDNA of B156 and B3. There was no nucleotide variation detected within the promoter region, whereas in the coding region, only one SNP mutation (G to T) was detected in B156, located within the second exon, 1693 bp downstream of the start codon, corresponding to the 485th nucleotide in the CDS (Fig. 2B). This mutation resulted in a W-to-L transition at the 162nd amino acid of the deduced protein sequence. Protein sequence analyses using BoCER2 and its close relatives revealed that the mutation site was adjacent to the HXXXD motif and was conserved in these species (Fig. 3). These results suggested that the 485th mutation might disrupt the function of *BoCER2* and might be responsible for the glossy trait of B156.

### Expression analysis of *BoCER2*

We performed qRT-PCR to reveal the expression pattern of *BoCER2* in wild-type B3 and mutant B156 plants. The results showed that *BoCER2* was expressed in the stems, leaves and siliques but was not expressed in the roots (Fig. 2C). By comparing the expression levels of *BoCER2* between B3 and B156. The results showed that the expression of *BoCER2* was significantly downregulated in the leaves, stems and siliques of the B156 mutant (Fig. 2C).

### Ectopic expression of *BoCER2* rescued the phenotype of *Arabidopsis cer2* mutant

**Fig. 4:**
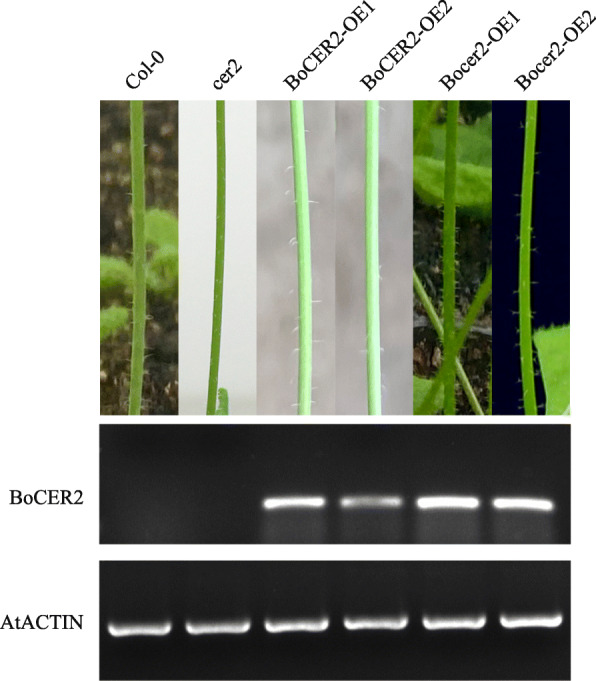
Functional analysis of *BoCER2* (from broccoli B3) and *Bocer2* (from broccoli B156) in the background of *Arabidopsis cer2* mutant. *BoCER2*-overexpression lines restored to wild type glaucous appearance, whereas *Bocer2-*overexpression lines still showed *cer2* glossy appearance. RT-PCR results of *BoCER2* and *AtACTIN* in Col-0 wild type plants, *cer2* mutant and transgenic plants are shown as cropped gels, and full-length gels are presented in Fig. S2

**Fig. 5:**
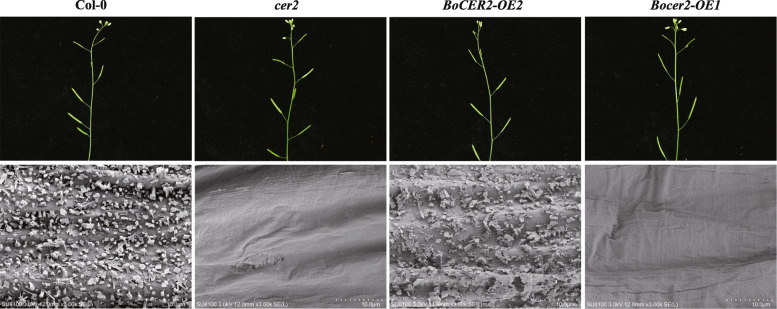
Phenotype of Arabidopsis stems and siliques, and scanning electron microscopy images of wax crystals on the surface of Arabidopsis stems. The Col-0 wild type and *BoCER2-OE1* plants are covered with wax crystals. The *cer2* mutant and *Bocer2*-OE1 plants lack wax crystals

We failed to introduce the *BoCER2* from B3 to the B156 mutant, because this plant material was recalcitrant to *Agrobacterium*-mediated transformation. Therefore, the function of *BoCER2* was tested in *A. thaliana cer2* mutant. We obtained 16 *BoCER2*-overexpression lines and 14 *Bocer2*-overexpression lines in the background of *Arabidopsis cer2* mutant. The phenotype and wax load of *BoCER2*-overexpression lines restored to wild type levels, whereas the *Bocer2*-overexpression still showed glossy appearance and lacking of cuticular waxes (Figs. 4 and 5), indicating that the broccoli *BoCER2* is a functional gene responsible for wax biosynthesis, and *Bocer2* is a loss-of-function allele.

## Discussion

The surfaces of terrestrial plants are covered by cuticles providing them with glaucous appearance. In contrast to wild-type plants, many mutants with a glossy appearance and their corresponding genes have been identified in *A. thaliana*, Maize and rice, revealing defects in cuticular wax biosynthesis. In addition to these model plant species, some glossy mutants have also been identified in Brassica species, including cabbage [19–22], Chinese cabbage [23], and rapeseed [24]. In this study, we identified a novel broccoli mutant, B156, whose glossy phenotype was similar to that of previously reported *10Q-974gl*, *10Q-961*, *HUAYOU2* and *LD10GL* cabbage lines [19–22]. Fine mapping, gene sequencing, gene expression and functional analyses revealed that *BoCER2* was a candidate gene responsible for the glossy trait.

Although the HXXXD acyltransferase motif was previously reported not required for CER2 function [10], this study indicated that the conserved sequence around HXXXD motif was essential for the function of BoCER2. *Arabidopsis CER2* was identified as *At4g24510* [16–18], which encodes an endoplasmic reticulum-localized protein homologous to BAHD acyltransferase. Its biochemical function in C28 VLCFA elongation was indicated by an assay in yeast [10]. However, the catalytic acyltransferase motif of CER2 was not conserved and not required for function, which is very different from the catalytic mechanism of other members of the BAHD family [10]. In this study, a SNP mutation within *BoCER2* in B156 should result in a W-to-L transition at the 162nd amino acid of the deduced BoCER2 protein sequence, which might disrupt the function of BoCER2 and result in the glossy traits of B156. Sequence comparison and ectopic expression in *Arabidopsis* revealed that the 162nd amino acid is a conserved site essential for the function of BoCER2. Thus, B156 should be a useful material for further characterization of the catalytic mechanism of CER2-related proteins.

We found that broccoli *BoCER2* might have experienced functional and expression divergence in comparison with *Arabidopsis CER2*. The C29 aldehydes are increased in B156 mutant, which is opposite to Arabidopsis *cer2* mutant, indicating that *BoCER2* may not be responsible for VLCFA elongation of aldehydes. The expression divergence between the *BoCER2* and *Arabidopsis CER2* is obvious. *Arabidopsis CER2* is expressed in aerial plant tissues, with high levels in stems and siliques, and trace level in leaves, but not in roots [17, 18]. Its mutant *cer2* showed significantly decreased total wax load on the stem [10, 17, 18]. However, the composition and load of wax on the leaves had no difference, possibly due to the functional redundancy of CER2, CER2-LIKE1 and CER2-LIKE2 in the leaves [10]. *BoCER2* was expressed in aerial organs similar to that of *Arabidopsis CER2*. However, the *BoCER2* transcript was highest abundant in the leaves. Interestingly, in contrast to the *Arabidopsis cer2* mutant, the B156 mutant showed an obvious glossy appearance of its leaves, indicating the predominant role of *BoCER2* in waxes accumulation on the surface of leaves. Further studies on the homologies among *CER2*, *CER2-LIKE1* and CER2*-LIKE2* in broccoli should provide new insight into the expression and functional divergence of these genes and leaf wax biosynthesis in *B. oleracea*.

The B156 mutant and the cloned BoCER2 have significance in breeding *B. oleracea* varieties. The glossy green broccoli mutant is, although currently not preferred by customers at least in China, useful to cabbage, another subspecies of *B. oleracea*, because the leaf product of this type is fresh and tender, and thus preferred by some customers. So breeders can introduced the locus from B156 to cabbage, which would help to create new glossy green varieties without any penalties of male fertility (unlike the *Bocer1* cabbage mutant). Moreover, identification genes involved in cuticular wax biosynthesis is of significance because of their roles in protecting plants against biotic and abiotic stresses. We have proved *BoCER2* to be a functional gene responsible for cuticular wax biosynthesis. It can be chosen as a target, which can be enhanced for better resistance to biotic and abiotic stresses, or knocked out by CRISPR/Cas9 system for direct creation of glossy green materials.

## Conclusions

In summary, we conducted phenotype analyses and genetic mapping of a novel glossy green mutant B156 in broccoli. Comparing with the wild type line, B156 mutant showed 63.5% reduction in total wax load, and lacking waxes mostly longer than C28, indicating elongation of C28 VLCFA was blocked in this mutant. Inheritance analysis revealed that the glossy trait was controlled by a recessive locus that was mapped to a 94.1 kb interval on C01 chromosome. In the target interval, only *BolC1t01909H* (*BoCER2*) was identified as a candidate for *BoGL5*. *Bocer2* of B156 mutant harbored a key SNP mutation located in a conserved site, providing a useful material for further characterization of the catalytic mechanism of CER2-related proteins. Further function characterization in *A. thaliana cer2* mutant suggested that *BoCER2* is a functional gene responsible for wax biosynthesis, and *Bocer2* is a loss-of-function allele. This study lays a foundation for further characterization of *BoCER2* and its utilization in enhancing stress tolerance and breeding of special cultivars with glossy appearance in *B. oleracea*.

## Methods

### Plant materials

B156 is a spontaneous glossy broccoli mutant generated from a natural broccoli population in 2010 and maintained by selfing. Another inbred line, B3, was crossed with a B156 parent to produce F_1_ plants. An F_2_ population comprising 1708 individuals was generated from self-pollination of the F_1_ progeny. *Arabidopsis* Col-0 wild-type line was preserved in our research group, and *cer2* mutant (SALK_084443C) was ordered from AraShare (https://www.arashare.cn/). All the plant materials were owned by the Institute of Vegetables and Flowers, Chinese Academy of Agriculture Sciences (IVFCAAS, Beijing, China).

### Cuticular waxes loads and compositions detection by SEM and GC-MS

Fresh leaves were collected from 8-week-old plants of B3 and B156. Scanning electron microscopy (SEM) and gas chromatography–mass spectrometry (GC-MS) were conducted as described by Liu et al. [19] and Cao et al. [25]. The wax loads were the means of three repetitions as ng/cm2. Additionally, stems of *Arabidopsis thaliana* were collected from 4-week-old plants for SEM analyses.

### Whole-genome nucleotide variation calling and primer design

We performed whole-genome resequencing of the parents, B156 and B3, using the Illumina HiSeq 2500 platform (Illumina, Inc., San Diego, CA, USA). The resequencing data were mapped to the reference genome sequence of broccoli “HDEM” (http://www.genoscope.cns.fr/externe/plants/index.html) [28], and single-nucleotide polymorphism (SNP) and InDel variations between the parents were retrieved. According to a previous study [29], InDel primers evenly distributed across the 9 chromosomes were designed using Primer 3.

### DNA isolation and fine mapping of the *BoGL5* gene

Genomic DNA was extracted from young leaves using a modified cetyltrimethylammonium bromide (CTAB) protocol [30]. All primers were screened using the parents’ DNA as a template. Polymorphic markers between the parents were further screened by bulked segregant analysis (BSA) [30]. Generally, two DNA pools (10 glossy F_2_ individuals for the G pool and 10 nonglossy F_2_ individuals for the NG pool) were constructed to detect markers linked to *BoGL5*. More InDel primers were designed around the linked markers to search for more linked markers. All glossy F_2_ individuals were genotyped to detect recombinants, construct a linkage map and narrow the location of the target gene. Polymerase chain reaction (PCR) and polyacrylamide gel electrophoresis (PAGE) were performed according to the methods in a previous report [31].

### RNA isolation, cDNA synthesis and expression analysis of the candidate gene by real-time PCR

Total RNA was extracted from tissues of the roots, stems, leaves, and siliques of B156 and B3 plants using an RNAprep Pure Plant Kit (Tiangen, Beijing, China), and first-strand cDNA was synthesized using a PrimeScript 1st Strand cDNA Synthesis Kit (Tiangen, Beijing, China). The protocols were performed according to the manufacturer’s instructions.

Quantitative RT-PCR (qRT-PCR) was performed to compare the expression levels of *BoCER2* in the B3 and B156 tissues. The qRT-PCR mixture was prepared in conjunction with SYBR Premix Ex Taq II (Tli RNase H Plus; Takara, Dalian, China), and the amplification was performed on a CFX96 Touch Real-Time PCR Detection System (Bio-Rad, Hercules, CA, USA). All samples were assayed in triplicate.

The transcript abundance of *BoCER2* in the samples was calculated using the 2^−ΔΔCT^ method. The specific primers for *BoCER2* and *B. oleracea actin* are listed in Table S3.

### Gene amplification and sequence analyses of *BoCER2*

The reference sequence of *BoCER2* was acquired from the HDEM reference genome and unpublished RNA-seq data. Based on the reference sequence, primers were designed using Primer 5.0 software. The ~ 2 kb promoter and gene body region was amplified using genomic DNA and/or first-strand cDNA of B156 and B3 as templates. PCR was performed using Q5 High-Fidelity DNA Polymerase (NEB).

Sequences of *A. thaliana CER2* were downloaded from The Arabidopsis Information Resource (TAIR) database (https://www.arabidopsis.org/). BLASTP was used to query the deduced amino acid sequence of BoCER2 for its relatives in *B. rapa* and *B. napus*. The obtained proteins were aligned using DNAMAN 6.0 (Lynnon Biosoft, CA, San Ramon, USA).

### Plasmid construction and *Arabidopsis* transformation

The coding sequences of *BoCER2* and *Bocer2* were amplified from 1st Strand cDNA of B3 and B156 respectively. The purified PCR products were inserted into a modified binary vector pBWA(V) BS (reconstructed from pCAMBIA1301) [32], placed downstream of the constitutive 35S promoter. The constructs were transformed into competent *Agrobacterium tumefaciens* GV3101, and into *Arabidopsis cer2* mutant (SALK_084443C) using the floral dip method [33]. Seeds were screened for hygromycin resistance on MS (Murashige and Skoog) medium containing 30 mg/L of hygromycin, and were further confirmed by RT-PCR analysis.

## Supplementary Information

**Additional file 1: Table S1.** Information of location, sequence, genotype (identified according to the resequencing data), and polymorphisms of all the markers used for genetic mapping.

**Additional file 2: Table S2.** Genotypic and phenotypic data of the F_2_ population. The plants were firstly genotyped by Broc94 and Broc4 to detect recombinants, which were further genotyped by additional eight markers.

**Additional file 3: Table S3.** Primers used in this study for genetic mapping, gene amplification and RT-PCR.

**Additional file 4: Figure S1.** Field performance of B3, B156 and the F_2_ population generated from the parents.

**Additional file 5: Figure S2.** Full-length gels showing the RT-PCR results of *BoCER2* and *AtActin*. The corresponding cropped gels were shown in Fig. 4.

## Data Availability

The raw sequencing data in this study are available in the SRA database (BioProject accession number: PRJNA695138). These data can be accessed using following link: https://www.ncbi.nlm.nih.gov/bioproject/PRJNA695138.

## Abbreviations

SEM: Scanning electron microscopy
GC-MS: Chromatography-mass spectrometry
VLCFAs: Very long chain fatty acids
ER: Endoplasmic reticulum
FAE: Multienzymatic fatty acid elongase
CRISPR: Clustered regularly interspaced short palindromic repeats
SNP: Single-nucleotide polymorphism
InDel: Insertion or deletion polymorphism
qRT-PCR: Quantitative real-time polymerase chain reaction
BSA: Bulked segregant analysis
PAGE: Polyacrylamide gel electrophoresis
CTAB: Cetyltrimethylammonium bromide
