# Supplementary material for: Genetic mapping and candidate gene identification of BoGL5, a gene essential for cuticular wax biosynthesis in broccoli
Source: BMC Genomics. 2021 Nov 10;22:811. doi: 10.1186/s12864-021-08143-7 (PMC8582161; doi:10.1186/s12864-021-08143-7)
Supplement: Supplementary file 3 — Additional file 3: Table S3. Primers used in this study for genetic mapping, gene amplification and RT-PCR. [file 12864_2021_8143_MOESM3_ESM.docx]

**Supplementary Table 3** Primers used in this study for genetic mapping, gene amplification and RT-PCR.

| Primer name | Forward primer sequence(5'-3') | Reverse primer sequence(5'-3') |
| --- | --- | --- |
| Broc2 | GAATCCAAACGGCCATTCTA | ATCTTCATCACGCAGCTCCT |
| Broc3 | TGTTTTGCTCTCCCTCTCTTTC | CTCCGATCCCAAACTGCTT |
| Broc4 | TGTGGATTCTGTAGGCCTCTTT | TCAGACCAGAGAACACAAAGTGA |
| Broc94 | CGCAAGGGACTACCAGTCAT | CGTTCTGGCCTGATCATCTT |
| Broc98 | TGCAGCAAAGACAAGTACCTC | GTTCCAATCAAAACGCATGA |
| Broc100 | CCACACTCTCACAACAACAACA | AGCAAAGGCTGGTGAAGCTA |
| Broc106 | GTCTTCTACCTCGGCGACAC | TCCGAATATCACTCGCTTTTG |
| Broc107 | GCGTAGCGGTTCTGGTTTAG | CGTTGACGATCTCTTCGTGA |
| Broc100 | ACACGAACAGACAAAGCGTG | TCAAGTTCTGAGCCTCCAAAG |
| Broc110 | AAAGCTTTTTCGTTTTCATGG | CCATCTTAGCCACGGTTGAT |
| Broc111 | TCCTCTCTCTCGGCGAATTA | GGTGACCTGTTCCTCACGAT |
| Broc112 | ATGATGGGTCCCACTCTGAT | TCCGTCTTTAGCCACGTTTT |
| Broc113 | GCAATGATCTCAACTCCGAAA | AAGATCCGGTGCTAGTCTCG |
| Broc117 | CTGAGCAACGTCTTGCAGAT | TGAAGATGTCAAAAGGGAATCA |
| Broc118 | TGCTCTAATTAAGACTCGGCTCA | ATTTGGAGGGGCCTTATCAC |
| Broc123 | ACTGTTTCTCGGCGGTAG | GCTCATCCTGCTTTTGCT |
| BoCER2-pro | GAGGCATCCACTCACCAGCAG | CGTGTACCCTTGAAGAAGTAAACAGC |
| BoCER2-gene | ATGGAGGCAAGCCCAGTG | CATTATCATATTATTCAACTCAATCTTCAGCTTTGC |
| BoCER2-RT | TGACAGGCGAGAACAAGC | TCATAACACGGGAGGTCG |
| AtACTIN | TGCTGACCGTATGAGCAAAG  AGCCATC | CAGCATCATCACAAGCATCC |
