# Supplementary figures and images for: Genetic mapping and candidate gene identification of BoGL5, a gene essential for cuticular wax biosynthesis in broccoli
Source: BMC Genomics. 2021 Nov 10;22:811. doi: 10.1186/s12864-021-08143-7 (PMC8582161; doi:10.1186/s12864-021-08143-7)

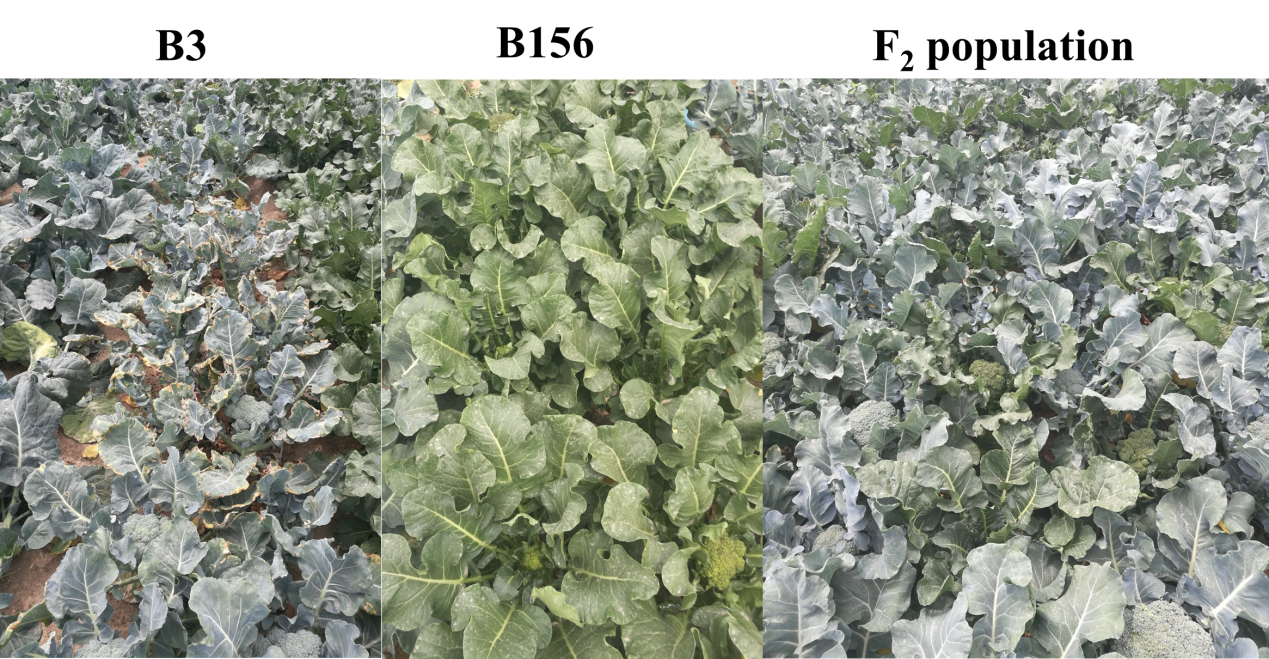


**Figure S1** Field performance of B3, B156 and the F2 population generated from the parents.

Supplement: Supplementary file 4 — Additional file 4: Figure S1. Field performance of B3, B156 and the F2 population generated from the parents. [file 12864_2021_8143_MOESM4_ESM.docx]
