# Supplementary material for: Genetic mapping and candidate gene identification of BoGL5, a gene essential for cuticular wax biosynthesis in broccoli
Source: BMC Genomics. 2021 Nov 10;22:811. doi: 10.1186/s12864-021-08143-7 (PMC8582161; doi:10.1186/s12864-021-08143-7)
Supplement: Supplementary file 5 — Additional file 5: Figure S2. Full-length gels showing the RT-PCR results of BoCER2 and AtActin. The corresponding cropped gels were shown in Fig. 4. [file 12864_2021_8143_MOESM5_ESM.docx]

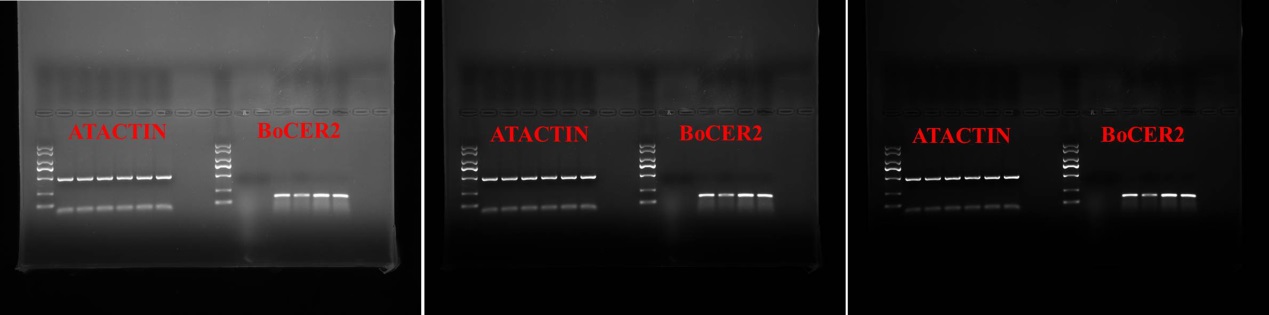


**Figure S2** Full-length gels showing the RT-PCR results of *BoCER2* and *AtActin*. The corresponding cropped gels were shown in Fig. 4.
